# Peroxisome protein import: a complex journey

**DOI:** 10.1042/BST20160036

**Published:** 2016-06-09

**Authors:** Alison Baker, Thomas Lanyon Hogg, Stuart L. Warriner

**Affiliations:** * School of Molecular and Cellular Biology, Astbury Centre for Structural Molecular Biology and Centre for Plant Sciences, University of Leeds, Leeds LS2 9JT, U.K.; † Institute of Chemical Biology, Department of Chemistry, Imperial College London, London SW7 2AZ, U.K.; ‡ School of Chemistry, Astbury Centre for Structural Molecular Biology, University of Leeds, Leeds LS2 9JT, U.K.

**Keywords:** mechanisms, models, peroxisome, PEX5, protein import cycle, targeting signal

## Abstract

The import of proteins into peroxisomes possesses many unusual features such as the ability to import folded proteins, and a surprising diversity of targeting signals with differing affinities that can be recognized by the same receptor. As understanding of the structure and function of many components of the protein import machinery has grown, an increasingly complex network of factors affecting each step of the import pathway has emerged. Structural studies have revealed the presence of additional interactions between cargo proteins and the PEX5 receptor that affect import potential, with a subtle network of cargo-induced conformational changes in PEX5 being involved in the import process. Biochemical studies have also indicated an interdependence of receptor–cargo import with release of unloaded receptor from the peroxisome. Here, we provide an update on recent literature concerning mechanisms of protein import into peroxisomes.

## Introduction

Peroxisomes are organelles found in all but the most primitive eukaryotic cells. They are surrounded by a single bilayer membrane which encloses a soluble matrix space. Peroxisomes contain no DNA and therefore all their constituent matrix proteins are imported post-translationally from the cytosol [1,2]. Peroxisome membrane proteins may be imported post-translationally, or in some cases after import into the endoplasmic reticulum followed by subsequent sorting pathways [3]. Peroxisomes are also able to divide and segregate to daughter cells upon cell division [4,5]. The list of peroxisome functions is long and ever growing. Peroxisomes have many different functions depending on cell type and environment, but fatty acid beta-oxidation and reactive oxygen species metabolism are common to most peroxisome types [6,7]. Peroxisomes can change their function by virtue of importing different proteins and enzymes [8]. The importance of peroxisomes for the proper development of multicellular organisms is underscored by the severe and frequently lethal phenotypes of both animal [9] and plant [10] peroxisome biogenesis mutants. Indeed, in humans the peroxisome biogenesis disorders have provided a strong motivation to study the underlying biochemistry, genetics and cell biology of peroxisomes [7].

The import of proteins into peroxisomes is quite different from the targeting and transport of proteins across other cellular membranes. This brief review provides an update on models and mechanisms of protein import into peroxisomes, focusing on the major matrix protein import pathway mediated by the import receptor PEX5.

## Molecular recognition of the PTS1 by PEX5

The first peroxisome targeting signal comprising of a C-terminal tripeptide serine–lysine–leucine (SKL in the single letter amino acid code) was discovered in firefly luciferase and termed peroxisome targeting signal 1 (PTS1) [11]. It was soon realized that PTS1 was conserved between organisms [12] and the discovery of its receptor protein, subsequently termed PEX5, rapidly followed [13–16]. Comparison of the sequences of native peroxisomal proteins, along with mutational analysis coupled with targeting studies and assessment of PEX5 binding preferences, established that the PTS1 is in fact a family of sequences which generally conform to the pattern of [small side chain amino acid]–[basic amino acid]–[hydrophobic amino acid], but in some cases can be considerably more diverse [17] and hinted that residues immediately adjacent to the C-terminal tripeptide might have an auxiliary function [18–20].

PEX5 is a modular protein which shows conservation of its essential features between organisms. The C-terminal domain comprises of seven tetratricopeptide (TPR) repeats. These are repeats of 34 amino acids that form a pair of helices that are a common protein–protein interaction motif [21] and bind the PTS1 peptide. Determination of the X-ray structure of the C-terminal domain of PEX5 from human [22] and *Trypanosoma brucei* [23] revealed molecular details of the interaction between the PEX5 TPR domain and model PTS1 peptides. The PTS1 binds within a cavity formed by two sets of TPRs, 1–3 and 5–7, with TPR4 adopting a more extended conformation linking the two sets of three TPRs. This provides a funnel shaped binding site in which the PTS1 peptide sits. A series of highly conserved asparagine residues within the TPRs make hydrogen bonds to the peptide backbone of the PTS1 sequence. The side chain in position -2 (lysine) of the model peptide, containing the C-terminal tripeptide SKL, sits within a negatively charged pocket where it hydrogen bonds via a water molecule. Serine in position -3 also makes hydrogen bonds to the receptor indirectly via water, as does the terminal carboxyl group. The side chain of the terminal leucine is accommodated in a hydrophobic pocket. These features explain why PTS1 must be at the C-terminus of the protein and the strong preference for a hydrophobic C-terminal residue, along with the lack of specific side-chain interactions which may allow adaptability in the recognition of sequence variants.

Structures of the PEX5 TPR domain in complex with whole proteins rather than just model peptides have given insights into contacts outside the PTS1. For both PEX5:sterol carrier protein 2 (SCP2), which has a consensus PTS1 AKL [24], and PEX5:alanine glyoxylate aminotransferase (AGT), which has the non-consensus PTS1 KKL [25], contacts between receptor and cargo protein extend beyond the PTS region; however, these differ between the two structures. In the SCP2:PEX5 complex this region of secondary contact to the receptor was distant from the PTS1 within the primary sequence [24] whereas in AGT the region immediately preceding the PTS1 makes extended contacts to the surface of PEX5 and mutations within this region result in a 2–5-fold reduction in affinity [25]. Mis-targeting of AGT to mitochondria due to mutations that increase mitochondrial targeting propensity and destabilize protein structure gives rise to the disorder primary hyperoxaluria type 1. The binding of variant AGT proteins and terminal octapeptides to the TPR domain of PEX5 was compared using isothermal titration calorimetry (ITC), molecular modelling and protein stability assays [26]. The authors concluded that a consensus PTS1 sequence increased the binding affinity principally by reducing the enthalpic penalty of binding, probably reflecting optimization of binding interactions through changes in conformation and buried surface area. The corresponding PTS1 peptides showed a similar order of binding affinities, but with approximately 10-fold weaker interaction, consistent with the idea that the C-terminus provides the main specificity but that other ancillary regions also contribute to the affinity. They also demonstrated that binding of peptides to PEX5 stabilized the receptor to thermal denaturation and proteolysis with the extent of protection correlating well with the measured binding affinity [26]. Direct evidence for structural reorganization of the receptor upon binding cargoes has been presented. For example the apo conformation of the PEX5 TPR domain is snail shaped and undergoes a conformational change to a ring shape upon binding SCP2 [24]. In the case of AGT binding to PEX5, mutation of the KKL targeting sequence to the consensus AKL removes a steric clash allowing the invariant PEX5 asparagine 534 to specifically bind to the -3 position of the PTS1. In turn this results in movement, especially of TPR6, leading to compaction of the PTS1 binding cavity [27]. By comparing the known PEX5:cargo protein structures it was proposed that high affinity binding correlates with a greater degree of compaction of the PTS1 binding pocket, and although less optimal PTS1 sequences are recognized, they are bound with lower affinity [27].

A site-directed photo-crosslinking approach was used to map interactions between *Saccharomyces cerevisiae* PEX5 and a peroxisomal oxalyl CoA synthetase (PCS60) which contains a consensus PTS1, SKL [28]. Crosslinks were formed predominantly between amino acids immediately preceding the PTS1 and TPRs 6, 7 and the 7C loop that is located C-terminal to TPR7. Placement of crosslinkers within the PTS1 tripeptide blocked interaction with PEX5, presumably through steric interference. Recombinant PCS60 binds PEX5 with a *K*_d_ of 0.19 μM as measured by ITC, but a variant in which the PTS1 was deleted was still able to bind with a measured *K*_d_ of 7.7 μM, even though this PTS1 deletion mutant could not import into peroxisomes *in vivo*. Using SPR, two binding events of wild-type PCS60 to PEX5 with different affinities could be detected. The high affinity binding event required the PTS1 sequence but the low affinity binding event was independent of the PTS1 and required the adjacent five residues. These results led to the proposal that interaction between PCS60 and PEX5 is a two-step process with an initial binding interaction followed by a ‘lock in’ [28].

**Figure 1:**
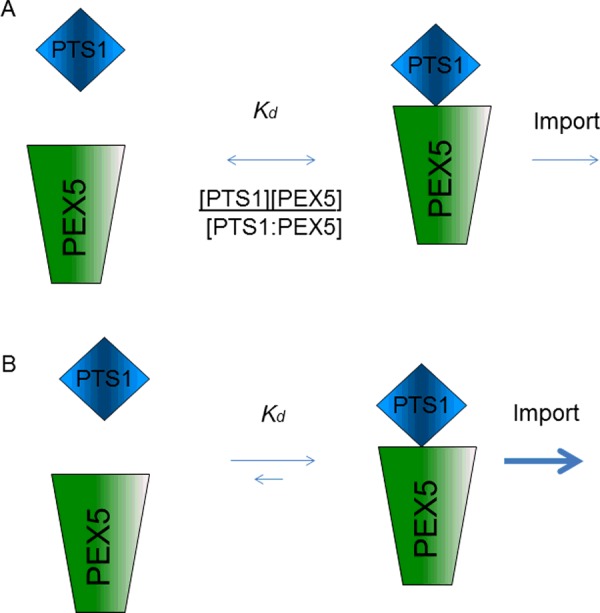
Models of PEX5:cargo interaction regulating import (**A**) In an equilibrium binding model the respective concentrations of PTS1 cargo protein [PTS1] and PEX5 receptor [PEX5] will determine the concentration of the receptor–cargo complex [PEX5:PTS1]. If PEX5:PTS1 must form for PTS1 cargo to be imported, any PTS1 cargo with *K*_d_ above the concentration of the receptor will not be imported. This model is not consistent with observed import of proteins *in vivo* which have *K*_d_ > 100 μM *in vitro* [32]. (**B**) If the PEX5:PTS1 complex is rapidly imported after formation, [PEX5:PTS1] in the cytosol will be kept low thus continually driving the equilibrium towards further formation of complex.

Binding affinities of model peptides to the PEX5 TPR have been determined in several studies and cover several orders of magnitude [29–31]. It was proposed that proteins with low levels of expression may have evolved stronger PTS's to compensate for their lower abundance [29]; however, it is difficult if not impossible to obtain accurate measurements of the cytosolic abundance of peroxisome proteins prior to import. Our recent study compared *in vivo* targeting, *in silico* targeting predictions and quantitative *in vitro* targeting peptide binding to PEX5 [32]. Although all three methods agreed well in their rank order of prediction, import *in vivo* was possible with sequences that were below the detection limit (*K*_d_ >100 μM) for binding to PEX5 *in vitro*. It is hard to imagine that either PEX5 or cargo proteins could exist at these concentrations *in vivo*, leading us to propose that import may not be a simple pre-equilibrium model where steady state concentrations of cargo and receptor determine import [32]. If downstream steps leading to import are rapid and irreversible, the effective concentration of receptor–cargo complex will be kept low, therefore driving the equilibrium towards formation of the receptor–cargo complex (Figure 1). Thus a protein that associates with the receptor may be ‘captured’ and committed to the import pathway even if it might otherwise rapidly dissociate from the receptor. The *in vivo* data demonstrate that the weak sequences are imported much more slowly than the strong ones, which suggests that only a small number of such molecules succeed in entering the import pathway at any one time [32]. Of course other factors may also come into play with native proteins such as auxiliary binding sequences, interactions with other cellular components such as chaperones, or in the case of peroxisomal proteins that assemble as oligomers in the cytosol, multivalent interactions leading to cooperativity of binding.

## The role of the N terminus of PEX5 in protein import

The N-terminus of PEX5 is believed to be a natively unstructured domain [33] and contains a number of functionally significant motifs. In humans and plants PEX5 contains the binding site for PEX7, which is the import receptor for a second class of matrix proteins that carry the N-terminal PTS2 signal [34,35]. PEX5 also contains several lysines and a conserved cysteine residue that are targets for ubiquitination. This modification regulates receptor turnover and recycling [36].

PEX5 from all organisms has multiple repeats of a sequence WX_3_F/Y which bind to PEX14, the docking factor for PEX5 at the peroxisome membrane [37–39]. Human PEX5 also contains an LVXE/F motif which binds PEX14 but dissociates over 30 times more rapidly, and is essential for import [40]. The role of the multiple PEX14 binding motifs is still unclear, but may have an important role in translocation. For example models in which a single PEX14 binds and slides along the PEX5 N-terminus, or in which multiple molecules of PEX14 are recruited to form the import pore have been proposed [40,41].

**Figure 2:**
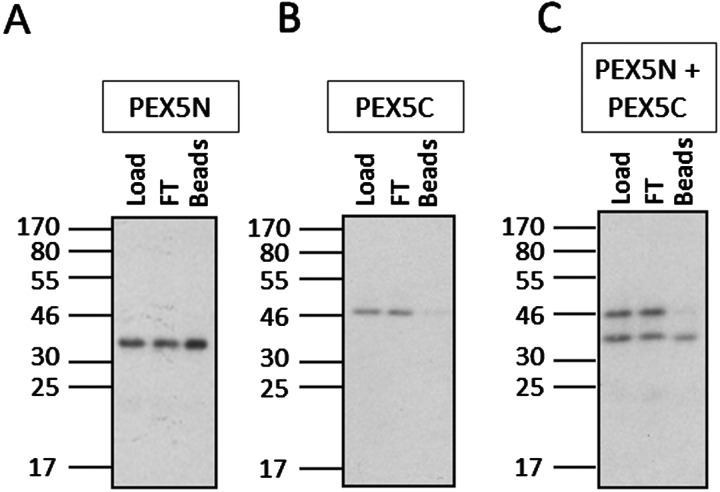
The N- and C-terminal domains of *Arabidopsis thaliana* PEX5 do not interact with one another in the absence of bound cargo Recombinant truncated PEX5 constructs, PEX5(1–304) (PEX5N) and PEX5(340–728) (PEX5C) both containing hexahistine tags were purified from *Escherichia coli* (see Supporting Information) and immunoprecipitated as follows. (**A**) PEX5N, α-PEX5N [69] positive control showing that PEX5N is immunoprecipitated. (**B**) PEX5C, α-PEX5N negative control showing that PEX5C is not immunoprecipitated. (**C**) PEX5N, PEX5C, α-PEX5N showing that PEX5N is immunoprecipitated but PEX5C is not co-immunoprecipitated, therefore the two domains of the protein do not interact. Proteins were incubated with antibody for 1 h before bound proteins were isolated from solution via protein A coupled beads. Load, sample of protein mixture; FT, sample of unbound proteins; Beads, immune isolated proteins.

Whether the N-terminal domain of PEX5 has a role in cargo binding is unclear. Some reports have indicated contacts between ‘atypical’ cargo proteins such as catalase and *S. cerevisiae* acyl CoA oxidase with PEX5 that lie outside of the TPR domain [42–44]. It has also been proposed that cargo binding causes a conformational change in PEX5 that releases the N-terminus for interaction with PEX14 [45]. The human PEX5 N526K mutant mimics the cargo bound state and therefore is a substrate for import [46]. It was further reported that the N- and C-terminal domains of PEX5 interact with one another in the absence of cargo [47]; however small angle X-ray scattering indicates that cargo-free PEX5 has an extended N terminal region that is free to bind PEX14 in a 1:6 ratio [48], and pull-down assays with recombinant proteins also show cargo-free interaction between PEX5 and PEX14 [49]. A lack of interaction of the N-terminal domain of PEX5 and the C-terminal TPR domain is also demonstrated by co-immunoprecipitation (Figure 2 and Supporting Information). Thus, whereas cargo binding clearly causes conformational changes within the PEX5 receptor, an increasing body of evidence disputes a model where cargo binding results in exposure of the N-terminus for interaction with PEX14.

## Mechanisms of translocation

Peroxisomes are unusual among protein translocation systems in that they have the capacity to translocate folded, even oligomeric proteins [50,51]; however, recent evidence supports the notion that in many cases monomers may be the preferred clients of the import machinery [52] and indeed PEX5 may actively inhibit oligomerization of some proteins [43]. Nevertheless there are some clearly documented examples of proteins that lack a PTS ‘hitch hiking’ into peroxisomes via interaction with a protein partner that does contain a PTS [53].

The observations that peroxisomes transport folded proteins implies that the translocation machinery can accommodate a wide variety of sizes and shapes. Cargo-loaded PEX5 itself enters the peroxisomal membrane in an ATP independent manner with the C-terminal TPR domain protected from externally added protease [54]. As discussed in the previous section, the N-terminus of PEX5 binds multiple molecules of PEX14 and a purified complex containing only PEX5 and PEX14 can be reconstituted to form a gated ion conducting channel, which is opened by presentation of a PEX5:cargo complex [55]. How cargo is unloaded from PEX5 remains unresolved. The atypical cargo protein catalase is released from PEX5 by binding of PEX14 to the most C-terminal WX_3_F/Y repeat [43], but whether PEX14 can act as a general PTS1 cargo unloader is not clear. PEX14 binding to PEX5 could not displace a PTS1 model peptide [49], but as additional contacts between PEX5 and some cargo proteins have been reported this experimental set up may not be wholly representative of PEX5 loaded with a native protein. What is clear is that cargo unloading takes place before receptor ubiquitination [54], described in the next section. The retention within the translocation machinery of a PEX5 construct fused to a bulky C-terminal tag has also been invoked as evidence that cargo unloading is a prerequisite for receptor recycling [56].

## Export coupled import

**Figure 3:**
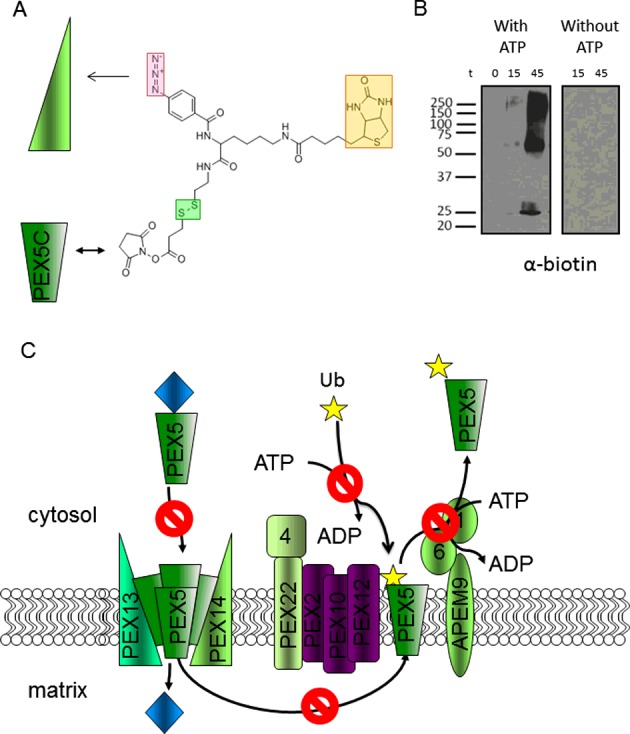
Label transfer approach to detect interactions with the import machinery (**A**) Sulfo-SBED is a trifunctional reagent with an amine reactive sulfo-NHS group which was used for coupling to PEX5C. Sulfo-SBED contains an aryl azide (pink box) which upon UV irradiation covalently links to a nearby ‘prey’ protein (green triangle), a biotin moiety for detection (yellow) and a cleavable disulfide (green) to allow release of the bait and transfer of the biotin label to the prey. (**B**) Western blot of carbonate extracted peroxisome membranes probed with streptavidin-HRP after an import reaction carried out in the presence or absence of ATP followed by UV irradiation. The time import was allowed to proceed for prior to UV irradiation is indicated above lanes in minutes. (**C**) Schematic diagram of the import machinery illustrating how, in the absence of ATP, ubiquitination of PEX5 could not take place. This would prevent PEX5 export and hence lead to its accumulation in the peroxisome membrane, thus preventing cargo-loaded PEX5 from inserting, even though the insertion step itself is not ATP-dependent. Blue diamond=PTS1 cargo. PEX14, PEX13; components of the docking apparatus on the peroxisome membrane. PEX22/PEX4; ubiquitin E2 ubiquitin ligase. PEX2/PEX10/PEX12; E3 ligase complex. Yellow star; ubiquitin. APEM9/PEX1/PEX6 receptor export complex (adapted from [65]: Bhogal, M.S., Lanyon-Hogg, T., Johnston, K.A., Warriner, S.L. and Baker, A. (2016) Covalent label transfer between peroxisomal importomer components reveals export-driven import interactions. J. Biol. Chem. **291**, 2460–2468).

Protein import into peroxisomes is a cyclical process. Cargo binds the receptor in the cytosol, docks at the peroxisome membrane, is unloaded and the receptor is recycled for further rounds of import [57]. Receptor recycling requires the addition of ubiquitin to a conserved cysteine residue near the N-terminus of PEX5 by a peroxisome localized E3 ligase complex [58] followed by export of the ubiquitinated receptor by the AAA ATPase complex [59]. These are the two ATP-dependent steps of the overall import process. Many years ago experiments with peroxisome *in vitro* import systems pointed to an ATP dependence of protein import into peroxisomes [60,61], which subsequently appeared inconsistent with results demonstrating that import is driven solely by thermodynamically favourable binding events [54,62]. Better understanding of the receptor export cycle has led to an appreciation that the import and export processes must be coupled, and that the ATP-dependent export process is necessary to remove PEX5 from the translocation pore [63] to facilitate further rounds of import. Mutation of the cysteine required for ubiquitination of *S. cerevisiae* PEX5 inhibits receptor export and blocks subsequent import which is consistent with this proposal [64]. A recently developed methodology in our laboratory that allows interrogation of protein–protein interactions during *in vitro* import of proteins into peroxisomes has also provided evidence consistent with export-coupled import [65]. In this method a bait protein is covalently labelled with a biotinylated, photo-activatable and thiol-cleavable crosslinker (sulfo-SBED) (Figure 3A). Upon incubation of a sulfo-SBED derivatized PEX5 construct with peroxisomes under conditions compatible with protein import, subsequent UV irradiation forms crosslinks to adjacent proteins. Reduction of the disulfide bond within the crosslinker transfers the biotin label to the prey protein(s) allowing for detection. Using this method we were able to demonstrate specific label transfer from an N-terminally truncated PEX5 construct (which would not be capable of recycling) to a heterogeneous complex of proteins in the peroxisome membrane. This labelling was blocked by inclusion of competing PEX14 or by the omission of ATP (Figure 3B). The peroxisomes are isolated under conditions that would be expected to promote association of endogenous PEX5 with the import pore, namely low temperature and without ATP [66], thus the import pore is probably fully occupied with endogenous PEX5 and prevents further insertion of labelled PEX5. In the presence of ATP endogenous PEX5 can be released from the pore, allowing insertion of labelled PEX5 and label transfer to the peroxisomal translocation machinery [65] (Figure 3C). The concept of coupling import and export has also been examined using a modelling approach, which supports the notion that export of one PEX5 molecule requires its replacement by a second incoming molecule [67].

## Conclusions and future directions

Recent years have seen considerable progress in understanding of the mechanism of peroxisomal protein import. In particular, the application of biochemical approaches has been fundamental in identifying and testing components and breaking down the import process into discrete steps. As understanding of individual steps within the peroxisomal import cycle has grown, it has become clear that a range of factors influence many of these processes. For example, ancillary, non-PTS1 interactions with PEX5 influence PTS1 cargo import, PEX14 displays the ability to provide both docking and unloading functions, and import steps have been linked to concomitant export steps in the translocation cycle. The interplay of these factors adds additional layers of complexity to the interpretation of experimental results, and requires increasingly intricate experimental design to unravel such effects. The study of the peroxisomal import machinery in a holistic manner therefore presents an unprecedented challenge; however, the continuing development of new analytical tools to build on current understanding offers increased promise in this endeavour. As progress in understanding the structures of component proteins at an atomic level provides new insights into molecular recognition, the logical development is into the structural study of larger complexes. New label transfer methods to detect interaction with the translocation machinery also afford the opportunity to quantify inhibition of such interactions by various factors, through Western blotting followed by densitometric analysis to generate IC_50_ curves. Application of computational approaches also has the potential to bring new insights into mechanism, but they require quantitative experimental data for model building and validation. It must also be remembered that all these biochemical processes take place in a complex cellular environment where activities are regulated in response to intrinsic and extrinsic signals, so approaches such as optical control over protein import are potentially interesting [68]. New methods and technologies will also drive further innovation for example developments in super resolution imaging that may one day allow us to ‘see’ the import process.

## Abbreviations

AGT: alanine glyoxylate aminotransferase
ITC: isothermal titration calorimetry
PTS1: peroxisome targeting signal 1
SCP2: sterol carrier protein 2
TPR: tetratricopeptide repeat
